# Guideline-Based Digital Exercise Interventions for Reducing Body Weight and Fat and Promoting Physical Activity in Adults With Overweight and Obesity: Systematic Review and Meta-Analysis

**DOI:** 10.2196/73656

**Published:** 2025-08-07

**Authors:** Mohamad Motevalli, Clemens Drenowatz, Derrick Tanous, Gerhard Ruedl, Werner Kirschner, Markus Schauer, Thomas Rosemann, Katharina Wirnitzer

**Affiliations:** 1 Department of Sport Science University of Innsbruck Innsbruck Austria; 2 Department of Secondary Education University College of Teacher Education Tyrol Innsbruck Austria; 3 Division of Sport, Physical Activity and Health University of Teacher Education Upper Austria Linz Austria; 4 Institute of Primary Care University of Zurich Zurich Switzerland; 5 Department of Pediatric Oncology and Hematology Charité – University of Medicine Berlin Berlin Germany; 6 Charité Competence Center for Traditional and Integrative Medicine Charité – University of Medicine Berlin Berlin Germany

**Keywords:** technology, mobile health, mHealth, eHealth, ubiquitous Health, uHealth, smartphone, weight loss, body composition, adulthood, training, fitness

## Abstract

**Background:**

Digitally delivered physical exercise interventions are becoming increasingly popular in addressing the obesity epidemic. However, there remains uncertainty on their efficacy regarding the reduction of body weight (BW) and body fat, which may, at least partly, be due to variations in study designs and inconsistent adherence to international physical activity (PA) guidelines.

**Objective:**

This study aimed to evaluate the effectiveness of digital exercise interventions based on PA guidelines in reducing BW and fat in adults with overweight or obesity, as well as their impact on PA-related factors.

**Methods:**

This review was conducted following the PRISMA (Preferred Reporting Items for Systematic Reviews and Meta-Analyses) guidelines. Comprehensive searches were performed in October 2024 across PubMed, Cochrane Library, Web of Science, and Ovid MEDLINE databases. Eligible studies included adults (aged ≥18 years) with objectively confirmed overweight or obesity who used digital interventions aligned with international PA guidelines. Risk of bias was evaluated using the Cochrane Risk of Bias (version 2) tool for randomized controlled trials and the Risk of Bias in Nonrandomized Studies of Interventions tool for nonrandomized studies. A random-effects meta-analysis with Hartung-Knapp adjustment was performed using R software.

**Results:**

Out of 4948 studies identified, 188 (3.8%) were screened in full and 30 (0.6%) met the eligibility criteria. Intervention durations ranged from 8 weeks to 24 months (average 6.4, SD 5.5 months). Meta-analysis showed that guideline-based digital exercise interventions significantly reduced BW compared to controls (mean difference [MD]=−1.17 kg; *P*=.003; *I*^2^=0.0%), with subgroup analysis revealing greater effects in active (nondigital) controls (MD=−1.23 kg; *I*^2^=7.5%) compared to passive (waitlist) controls (MD=−0.52 kg; *I*^2^=0.0%). A significant reduction in BMI was observed (MD=−0.50 kg/m^2^; *P*=.003), although with substantial heterogeneity (*I*^2^=70.0%), and subgroup analysis showed greater effects compared to passive controls (MD=−0.70 kg/m^2^; *I*^2^=43.1%) rather than to active controls (MD=−0.45 kg/m^2^; *I*^2^=74.5%). No significant effect was observed for body fat percentage overall (MD=−0.08%; *P*=.84; *I*^2^=7.4%). Qualitative analysis (including findings from noncomparative studies) showed that guideline-based digital exercise interventions led to significant reductions in BW (22/25, 88% studies; range −1.3 to −8.4 kg); BMI (19/23, 83% of studies; range −0.4 to −3.4 kg/m^2^); waist circumference (15/16, 94% of studies; range −2.1 to −9.2 cm), body fat percentage (9/9, 100% of studies; range −0.3% to −4.1%); and fat mass (7/7, 100% of studies; range −0.4 to −6.5 kg), while findings for waist-to-hip ratio and PA outcomes were inconsistent.

**Conclusions:**

Guideline-based digital PA and exercise interventions show potential in reducing excess BW in adults with overweight or obesity, with stronger effects when compared to nondigital interventions. However, their superiority over traditional methods is uncertain for BMI and body composition. Substantial variations in study designs present challenges in drawing definitive conclusions on specific characteristics of effective digital exercise tools.

**Trial Registration:**

PROSPERO CRD42024620020; https://www.crd.york.ac.uk/PROSPERO/view/CRD42024620020

## Introduction

### Background

Overweight and obesity are among the most pressing public health challenges of the 21st century, posing significant health risks and economic burdens worldwide [1]. According to the World Health Organization (WHO), the prevalence of adult obesity has more than doubled since 1990 [2]. By 2022, 43% of adults aged ≥18 years were classified as overweight, and 16% were living with obesity, making it a major risk factor for several chronic diseases, including cardiovascular disease, type 2 diabetes, musculoskeletal disorders, and cancer [2]. Data shows that being overweight or obese is associated with approximately 2.8 million deaths each year [3]. This highlights the urgent need for effective interventions to manage body weight (BW) and improve body composition in adults with excess body fat.

Physical activity (PA) is recognized as a critical component of obesity prevention and management strategies [4]. The WHO guidelines recommend that adults engage in at least 150 to 300 minutes of moderate-intensity aerobic activity or 75 to 150 minutes of vigorous-intensity activity per week [5]. It has been well documented that adherence to these recommendations is associated with significant improvements in body composition [6], emphasizing the critical role of PA in mitigating obesity-related health issues. Despite its proven benefits, adults often struggle to maintain sufficient levels of PA due to barriers such as time constraints, lack of access to fitness facilities, and motivation, highlighting the need for innovative solutions that make PA and exercise more accessible and sustainable [7].

Today’s digital world represents a transformative change in how we live, work, and interact [8], characterized by the integration of digital technologies into almost every aspect of human life. One of the most significant outcomes of this technological revolution is the expansion of mobile apps, which have become integral tools in daily life. An app is defined as software designed for specific tasks, which can be downloaded and installed on mobile phones or other digital devices [9]. In recent years, the advancement of digitalization has led to the emergence of digital health interventions as promising tools for supporting BW management and promoting healthy lifestyles [10]. Among these, PA and fitness apps have gained considerable attention by leveraging technological advancements to offer and deliver exercise plans, while also monitoring PA and fitness progress [11].

Over the past 2 decades, several studies have examined the effectiveness of health and fitness apps in BW management and other health outcomes [12-22]; however, the results have been inconsistent, with some studies reporting significant benefits while others found minimal or no effect on target outcomes. This variability in results may be attributed to differences in study design, population characteristics, app features, and intervention characteristics. More importantly, existing studies on digital PA and exercise interventions often lack a standardized implementation of key exercise characteristics (such as type, frequency, duration, and intensity) based on international PA guidelines. This inconsistency makes it difficult to compare interventions and assess their adequacy, thereby potentially influencing the outcomes. Therefore, a comprehensive review of digital PA interventions that are based on PA guidelines, such as those from the WHO or an equivalent framework, is needed to ensure more consistent and reliable outcomes.

### Objectives

The primary objective of this systematic review was to evaluate the effectiveness of digital exercise interventions that were based on international PA recommendations on body composition, as well as PA and fitness outcomes, in adults with overweight and obesity. In addition, while this review aimed to identify gaps in the literature, its overarching goals were to provide insights that inform clinical practice and public health initiatives, and to guide the design of future digital interventions, thereby contributing to the effective management of overweight and obesity on a broader scale.

## Methods

### Study Protocol

This systematic review and meta-analysis was conducted in accordance with the PRISMA (Preferred Reporting Items for Systematic Reviews and Meta-Analyses) guidelines [23]. The PRISMA checklist was used to guide the reporting across all stages of the review, encompassing the formulation of the research question, establishment of eligibility criteria, development of the search strategy, study selection, data extraction, and synthesis of results. The review protocol was registered prospectively in the PROSPERO under the registration number CRD42024620020. The protocol’s title was revised during the review process; however, no other deviations from the registered protocol occurred.

### Eligibility Criteria

The eligibility criteria encompassed multiple dimensions guided by the population, intervention, comparison, outcome, and study design framework [24], along with additional considerations. The population of interest comprised adults aged ≥18 years with overweight or obesity. Overweight and obesity were defined using established thresholds: a BMI ≥25.0 kg/m^2^ [25] or ≥23.0 kg/m^2^ for Asia-Pacific adult populations [26], body fat percentage (BF%) ≥25% for male individuals and ≥36% for female individuals [27], or a waist-to-hip ratio (WHR) ≥0.90 for male individuals and ≥0.80 for female individuals [28]. Baseline measures of BW status had to be objectively assessed; studies relying on self-reported anthropometric data were excluded. To ensure the feasibility of participation in PA and exercise interventions, studies were required to include participants without severe health conditions or physical limitations that could hinder PA and exercise engagement. Studies involving trained participants who had been regularly engaged in PA, exercise, or sports within the 3 months before the intervention were also excluded. Furthermore, studies were excluded if they included participants who were pregnant or within 1 year postpartum, as these conditions can significantly influence body composition.

Regarding the intervention criteria, the review targeted interventional studies assessing the effectiveness of digital PA and exercise programs designed in alignment with WHO guidelines on PA, that is, a minimum of 150 minutes of moderate-intensity PA or 75 minutes of vigorous-intensity PA per week [5], or a comparable framework. “PA” refers to any bodily movement produced by skeletal muscles that requires energy expenditure, while “exercise” or “physical exercise” is a subset of PA that is planned, structured, and repetitive, with the specific objective of achieving or maintaining health and fitness [29,30]. Eligible studies implemented PA or exercise interventions with a minimum duration of 8 weeks. In controlled trials, the control group (whether consisting of participants not engaging in physical exercise or those who did not engage in any digital interventions) was considered the comparator. For single-arm interventional studies, comparisons were made against baseline values of the same participants assessed before the intervention.

The primary outcomes of interest included anthropometric and body composition measures: BW, BMI, BF%, fat mass (FM), waist circumference (WC), and WHR. All outcomes had to be objectively measured; studies relying on self-reported data were excluded to minimize bias and ensure reliability. Any study that reported at least one of these outcomes was eligible for inclusion. The secondary outcomes included PA-related or physical fitness variables, regardless of the assessment method. Finally, eligible studies were restricted to publications in English or German, and no limitations were applied regarding the publication period, context, setting, or geographic location.

### Information Sources and Search Strategy

A comprehensive search strategy was used to identify relevant studies for inclusion in this systematic review, conducted from October 1 to 10, 2024. The databases searched included PubMed, Cochrane Library, Web of Science, and Ovid MEDLINE. The search strategy used a combination of keywords and Medical Subject Headings (MeSH) terms with the Boolean operators “AND” and “OR,” as detailed in Multimedia Appendix 1. In addition to the database searches, the reference lists of the included studies and relevant review articles were manually reviewed to identify any additional publications that may not have been captured in the database search.

### Study Selection and Data Extraction

The study selection process was conducted using Covidence, a specialized tool for systematic screening and data extraction. Two independent reviewers (MM and DT) screened the titles and abstracts of all retrieved articles based on the predefined eligibility criteria. Any discrepancies between the 2 reviewers were resolved through discussion. Articles deemed potentially relevant during the abstract screening phase underwent full-text review, and the same reviewers thoroughly assessed the full text of each article, while reasons for exclusion were documented. A third reviewer (CD) was consulted for the final decision if consensus could not be reached. Studies that met all the eligibility criteria during the full-text screening process advanced to the data extraction phase, where the same reviewers extracted the data. The extracted data encompassed several domains, including study characteristics, study design, intervention details, sample size, population, app or digital tool specifics, PA or exercise protocols, comparators, anthropometric and body composition outcomes, and PA and fitness outcomes. When the data were unclear, incomplete, or missing, the reviewers contacted the authors of the studies to request clarifications or additional information. When multiple articles reported the same study with identical population, outcomes, and data, the earliest publication was included to avoid data duplication. Any exclusions were carefully considered and documented to ensure transparency in the selection process.

### Risk of Bias Assessment

The risk of bias in the included studies was assessed using standardized tools to ensure a thorough evaluation of methodological quality. Two independent reviewers (MM and DT) assessed the risk of bias, and any discrepancies were resolved through discussion. For randomized controlled trials (RCTs), the Cochrane Risk of Bias (version 2) tool was used [31]. This tool evaluates bias across 5 key domains: randomization process, deviations from intended interventions, missing outcome data, measurement of the outcome, and selection of the reported result. Each domain is assessed for potential sources of bias that could impact the validity of the trial’s findings. The Risk of Bias in Nonrandomized Studies of Interventions tool was used for nonrandomized and single-arm studies [32]. This tool evaluates 7 domains: confounding, selection of participants, classification of interventions, deviations from intended interventions, missing data, outcome measurement, and selection of the reported result. Using both tools enabled a comprehensive assessment of the methodological rigor and potential biases in each study, ensuring a reliable interpretation of the evidence.

### Data Synthesis

A qualitative synthesis of the data was conducted, focusing on the various outcomes reported across studies. The extracted data were systematically grouped and analyzed by outcome categories and variables (study design, population, study group, app or digital tool, intervention, primary outcomes, and PA outcomes), enabling a detailed exploration of trends and patterns in the evidence. The synthesis aimed to identify consistent trends across studies while also highlighting areas of agreement, inconsistencies, or discrepancies in the findings. This approach provided a deeper understanding of the broader implications of the data and helped identify areas where further research is needed to resolve conflicting results or clarify uncertainties.

Meta-analysis was performed using R (version 4.5.0; R Foundation for Statistical Computing) to evaluate the effects of digital exercise interventions on primary outcome variables. A random-effects model with Hartung-Knapp adjustment was applied to account for potential between-study heterogeneity and to provide robust CIs. The primary effect size metric was the mean difference (MD) between intervention and control groups, expressed in the original units for each outcome. Subgroup analyses were conducted to examine potential differences in intervention effects by control type. Studies were stratified based on the type of control condition: (1) active controls (ie, nondigital interventions or usual care) and (2) passive controls (ie, waitlist—participants assigned to receive the intervention after the study period—or no intervention). When studies included multiple eligible intervention arms, each arm was treated as a separate unit of analysis to avoid statistical dependency, with the intervention arm serving as the unit of analysis. Heterogeneity was assessed using the *I*^2^ statistic, *τ*^2^, and Cochran Q test. All procedures were conducted following the PRISMA guidelines, and results are reported with 95% CIs and corresponding *P* values.

## Results

### Selection Process

**Figure 1:**
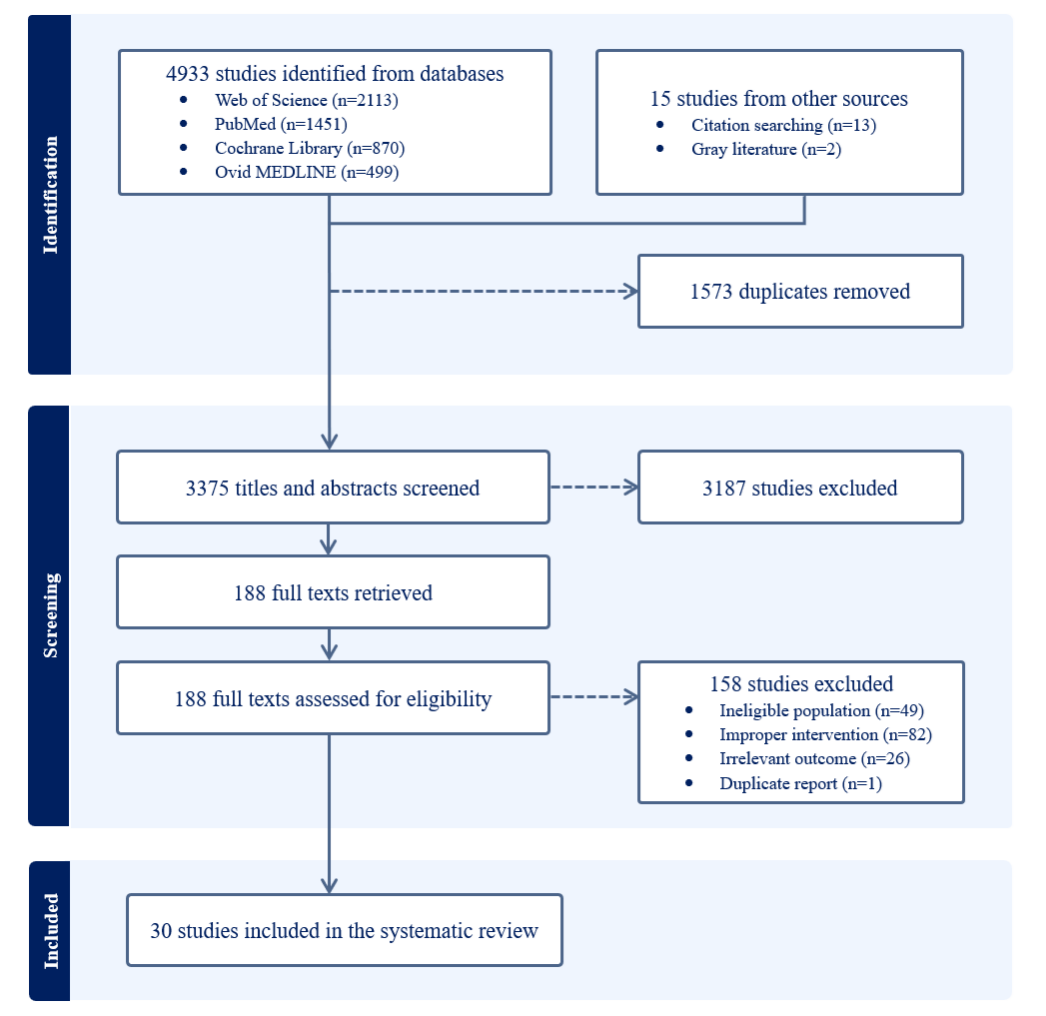
PRISMA (Preferred Reporting Items for Systematic Reviews and Meta-Analyses) flowchart showing the study identification, screening, and inclusion process.

A total of 4948 studies were initially identified through the databases and reference sources. After removing duplicates, 68.21% (3375/4948) of the studies were screened by title and abstract, of which 5.57% (188/3375) of the studies were retrieved for full-text review. Ultimately, 30 studies met the predefined eligibility criteria and were included in the systematic review. Figure 1 presents the PRISMA flowchart outlining the study selection process.

### Included Studies

The final sample of included studies consisted of 26 RCTs and 4 nonrandomized studies. Out of the 30 included studies, 11 (37%) were conducted in the United States, 3 (10%) in Australia, 3 (10%) in Korea, 2 (7%) in the United Kingdom, 2 (7%) in Germany, 2 (7%) in Spain, and 1 (3%) each in Sweden, France, Canada, Belgium, Singapore, China, and Hong Kong. The sample sizes varied from 16 to 750 participants, with an average of 132 participants. Of the 30 included studies, 6 (20%) exclusively included female participants, while none of the included studies focused solely on male participants. Table 1 provides a summary of the characteristics of the studies included in the review.

**Table 1:** Summary of the characteristics and outcomes of the studies included in the systematic review. The studies are ordered by intervention duration.

Study, year, and country			Design and population		Study groups		Intervention		Primary outcomes and findings		PA^a^ outcomes and findings
**Intervention duration: 24 months**											
	Jakicic et al [33], 2016, United States	Two-arm RCT^b^; sample size: 470; median age 30.9 years; 71.1% female participants; median BMI 31.2 kg/m^2^		Enhanced intervention (n=237) Standard intervention (n=233)		Digital tool: FIT Core; BodyMedia; a 24-month intervention where all participants initially increased PA, followed a low-calorie diet, and attended group counseling. At 6 months, telephone counseling, text message prompts, and website access were introduced. The standard group used a website for self-monitoring, while the enhanced group used a wearable device with a web interface. Moderate to vigorous PA progressed from 100 to 300 min per week over 4-week intervals.		Significant decreases in BW^c^ BMI, BF%^d^, and FM^e^ in both groups; no significant between-group differences for BMI, BF%, and FM, while postintervention BW was significantly lower in the standard group.		Significant improvements in LPA^f^ and MVPA^g^ in both groups; no significant between-group differences	
	Svetkey et al [34], 2015, United States	Three-arm RCT; sample size: 365; mean age 29.4 years; 69.6% female participants; mean BMI 35.2 kg/m^2^		mHealthh (n=123) Personal coaching (n=120) Control (n=122)		Digital tool: Investigator-designed app; a 24-month intervention delivered via a smartphone app based on social cognitive theory and transtheoretical model, incorporating goal setting, behavioral self-management, and motivational enhancement, with a PA target of achieving ≥180 min per week of moderate PA.		Significant differences in BW between the study groups, with greater reductions in the personal coaching group at 6 and 12 months, but not at 24 months.		—^i^	
**Intervention duration: 12 months**											
	Héroux et al [35], 2017, Canada	Single-arm intervention trial; sample size: 28; mean age 49.6 years; 100% female participants; mean BMI 32.2 kg/m^2^		Intervention (n=28)		Digital tool: The online Precision Nutrition Coaching Program; a 12-month online program focused on daily physical exercise, dietary habits, and health behavior lessons, with coaching delivered via computer, tablet, and mobile device. The exercise program included detailed daily workouts (aerobic interval training, weight training, or walking) with progressive intensity over time.		Significant decreases in BW, WC^j^, and FM (but not in BMI).		—	
	Lynch et al [36], 2017, United States	Single-arm intervention trial; sample size: 46; mean age 39 years; 65.2% female participants; mean BMI 31.8 kg/m^2^		Intervention (n=46)		Digital tool: Lean Eating by Precision Nutrition Coaching; a 12-month weight loss intervention with daily components of physical exercise, nutrition, behavioral modification strategies, and health lessons via an online platform, along with online coaching. The PA component included at least 150 min of physical exercise per week, with a minimum of 2 strength training sessions per week.		Significant decreases in BW, BMI, and BF% (but not in WHR^k^).		—	
	Watson et al [37], 2015, United Kingdom	Two-arm RCT; sample size: 65; mean age 52.2 years; 55.4% female participants; mean BMI 32.6 kg/m^2^		Web-based (n=32) Usual self-care (n=33)		Digital tool: Imperative Health; a 12-month web-based weight loss intervention incorporating PA and dietary components, along with goal setting and tailored feedback and support provided by physiologists via telephone and email. The PA program included a weekly schedule for planning activities at light to vigorous intensity levels.		Significant decreases in BW in the intervention group, with significant between-group differences at 3 and 6 months, but not at 12 months. Significant between-group differences in BMI and WC at 3 and 6 (but not at 12) months.		Significant between-group differences in daily PA at 3 months, but not at 6 and 12 months.	
**Intervention duration: 6 months**											
	Alcántara-Aragón et al [38], 2018, Spain	Two-arm RCT; sample size: 183; mean age 44.5 years; 83.6% female participants; mean BMI 34.75 kg/m^2^		Telematic intervention (n=91) Nontelematic intervention (n=92)		Digital tool: PREDIRCAM2 web platform; a 6-month tailored physical exercise and dietary plans based on international PA guidelines and the Mediterranean diet. The telematic group received online support and virtual contact, while the nontelematic group followed traditional methods.		Significant decreases in BW, WC, and WHR in the telematic intervention group; no significant between-group differences.		—	
	Allen et al [39], 2013, United States	Four-arm RCT; sample size: 68; mean age 44.9 years; 77.9% female participants; mean BMI 34.3 kg/m^2^		Intensive counseling (n=18) Smartphone only (n=17) Intensive counseling+smartphone (n=16) Less intensive counseling+smartphone (n=17)		Digital tool: Lose It!; a 6-month intervention (PA and diet) using an eclectic approach combining social cognitive theory, behavioral self-management, and motivational interviewing techniques to achieve 5% BW loss and a minimum of 150 minutes of moderate-intensity PA per week. Intensive groups attended frequent in-person sessions, while less intensive and smartphone-based groups had fewer sessions.		Significant decreases in BW, BMI, and WC in all groups; no significant between-group differences.		No significant between-group difference in MVPA.	
	Apiñaniz et al [40], 2019, Spain	Two-arm RCT; sample size: 110; mean age 38.5 years; 71.8% female participants; mean BMI 32.7 kg/m^2^		Health advice+app (n=54) Health advice (n=56)		Digital tool: AKTIDIET; a 6-month intervention consisting of health advice (PA and dietary) based on international guidelines for both groups. In the intervention group, this advice was reinforced through an app consisting of aerobic and muscle training programs and food intake tracking.		Significant decreases in BW in both groups; no significant between-group differences.		—	
	Batsis et al [41], 2021, United States	Single-arm intervention trial; sample size: 53; mean age 72.9 years; 69.8% female participants; mean BMI 36.5 kg/m^2^		Intervention (n=53)		Digital tool: Video conferencing; a 6-month weight management program combining synchronous videoconference-based physical exercise and nutrition sessions, remote Fitbit monitoring, and periodic face-to-face interactions. Participants attended 75-minute, twice-weekly group exercise sessions led by a trained physical therapist.		Significant decreases in BW, BMI, and WC; no change in WHR.		Significant improvements in 30-second sit-to-stand and 6-minute walk test; no changes in gait speed or grip strength.	
	Block et al [42], 2015, United States	Two-arm RCT; sample size: 339; mean age 55.0 years; 31.3% female participants; mean BMI 31.2 kg/m^2^		Alive-PD intervention (n=163) Usual care control (n=176)		Digital tool: Alive-PD; a 6-month program with weekly goal setting (PA and dietary) delivered via web, email, interactive voice response calls, and a supportive mobile app. The PA target included 150-300 minutes of aerobic activity and resistance training weekly, based on baseline levels and progress.		Significant decreases in BW, BMI, and WC in both groups, with significantly greater reductions in the intervention group.		—	
	Duncan et al [18], 2020, Australia	Three-arm RCT; sample size: 116; mean age 44.5 years; 70.7% female participants; mean BMI 31.7 kg/m^2^		Enhanced (n=39) Traditional (n=41) Waitlist control (n=36)		Digital tool: Balanced app; a 6-month multicomponent (PA and dietary) mHealth intervention delivered via a smartphone app, providing educational materials, goal setting, self-monitoring, and feedback; the enhanced group received additional sleep-related support. PA action planning focused on MVPA, resistance training, and step count increases.		No significant between-group difference in BW, while postintervention WC was significantly lower in the traditional group.		No significant between-group differences in MVPA and LPA.	
	Hartman et al [43], 2016, United States	Two-arm RCT; sample size: 55; mean age 59.5 years; 100% female participants; mean BMI 31.9 kg/m^2^		Technology-based (n=36) Usual care (n=18)		Digital tool: Fitbit and a website; a 6-month weight loss intervention focused on developing and practicing self-monitoring and self-regulatory skills, with a goal of losing 10% of BW by engaging in at least 150 minutes per week of MVPA and restricting calorie intake.		Significant decreases in BW in the intervention group, with a significant between-group difference (no BW change in the usual care group).		Significant improvement in daily MVPA in the intervention group, with no significant between-group differences.	
	Haufe et al [44], 2021, Germany	Two-arm RCT; sample size: 314; mean age 48.1 years; 14% female participants; mean BMI 33.3 kg/m^2^		Telemonitoring physical exercise (n=160) Waitlist control (n=154)		Digital tool: Custom-designed smartphone app; a 6-month telemonitoring-supported lifestyle intervention focused on regular physical exercise, including individual exercise recommendations provided during face-to-face meetings and via a smartphone app, aiming for at least 150 minutes of PA per week.		Significant decrease in WC in the intervention group, with a significant between-group difference (less but significant decrease in WC in the control group).		Significant increase in exercise capacity in both groups, with a significantly greater increase in the intervention group.	
	Hutchesson et al [20], 2018, Australia	Two-arm RCT; sample size 57; mean age 27.1 years; 100% female participants; mean BMI 29.4 kg/m^2^		Intervention (n=29) Control (n=28)		Digital tool: Be Positive Be Health e; a 6-month weight loss program delivered via eHealth technologies (website, app, email, text messages, and social media), incorporating goal setting and grounded in social cognitive theory and control theory, aligned with international PA and dietary guidelines.		Significant decreases in BW, BMI, FM, and BF% in the intervention group, with no significant between-group differences except for FM. Significant decreases in WC in both groups.		No significant changes in MVPA, MPA^l^, and VPA^m^ in any study group.	
	Lim et al [45], 2022, Singapore	Two-arm RCT; sample size: 148; mean age 53.1 years; 39.9% female participants; mean BMI 29.8 kg/m^2^		Intervention (n=72) Control (n=76)		Digital tool: nBuddy Diabetes app; a 6-month in-app dietitian coaching incorporating behavioral strategies (goal setting, stimulus control, problem-solving, self-monitoring, cognitive restructuring, and motivational interviewing), with a PA goal of 150 minutes per week of moderate-intensity physical exercise.		Significant decreases in BW and BMI in both groups, with significantly greater reductions in the intervention group.		Significant improvement in general PA in the intervention group; no significant between-group difference.	
	Rogers et al [46], 2016, United States	Three-arm RCT; sample size: 39; mean age 39.9 years; 79.5% female participants; mean BMI 39.5 kg/m^2^		Standard (n=14) Technology (n=12) Enhanced technology (n=13)		Digital tool: BodyMedia Fit and LINK; a 6-month mHealth intervention program incorporating PA and dietary components, alongside self-monitoring and feedback. The PA component involved unsupervised, home-based physical exercise at moderate intensity, with a gradual increase in session duration.		Significant decreases in BW, BMI, WC, BF%, and FM in all groups; no significant between-group differences.		—	
**Intervention duration: 12-16 weeks**											
	Hurst et al [47], 2021, United States	Single-arm intervention trial; sample size: 30; mean age 38.1 years; 43% female participants; median BMI 32.7 kg/m^2^		Intervention (n=30)		Digital tool: Telehealth coaching; 16-week telehealth intervention delivered in primary care clinics, using wearable devices, automated text messaging, and trained health coaching (including real-time feedback) to support physical exercise and nutrition goals, including achieving at least 150 minutes of PA per week.		Significant decreases in BW and BMI.		Significant improvement in MVPA.	
	Stephens et al [48], 2017, United States	Two-arm RCT; sample size: 62; median age 20.0 years; 71% female participants; median BMI 28.5 kg/m^2^		Smartphone + health coach (n=31) Control (n=31)		Digital tool: Lose it!; a 3-month intervention using a behavior-based smartphone app (primarily grounded in self-efficacy theory) for weight loss, combined with counseling sessions and text messages from a health coach, with goals of losing 1-2 pounds per week and engaging in at least 150 minutes of moderate-intensity PA weekly.		Significant decreases in BW, BMI, and WC in the intervention group (but not in the control group), with significant between-group differences.		Significant improvement in PA score in the intervention group, with no significant between-group difference.	
	Wong et al [49], 2021, Hong Kong	Two-arm RCT; sample size: 77; mean age 58.9 years; 55.8% female participants; mean BMI 27.02 kg/m^2^		App (n=38) Booklet (n=39)		Digital tool: MetS app; a 3-month lifestyle intervention delivered via a mobile app to support participants’ individual physical exercise routines through goal setting, logging exercise type and duration, and self-monitoring. The PA goal was at least 30 minutes per day, 5 days per week.		Significant decreases in BW and BMI (but not in WC or WHR) in the app group, with significant between-group differences for BW and BMI (no change in the booklet group).		Significant improvements in exercise score and exercise self-efficacy in the app group, with significant between-group differences.	
	Zhang et al [50], 2023, China	Three-arm RCT; sample size: 750; mean age 70.1 years; 53.9% female participants; mean BMI 27.7 kg/m^2^		Remote PA and diet (n=250) Remote PA (n=250) Control (n=250)		Digital tool: mHealth app; a 3-month remote weight management intervention focused on PA (with or without dietary components) including health assessments and guidance from exercise instructors and nutritional professionals. The PA program included 20 minutes of resistance or aerobic exercise or walking 6000 steps daily.		Significant decreases in BW, BMI, WC, and WHR in the remote PA and diet group, with significant between-group differences at day 90, but not at day 45.		No significant changes in total PA and PA levels in any study group.	
	Anderson et al [51], 2018, United Kingdom	Two-arm RCT; sample size: 78; mean age 47.1 years; 88% female participants; mean BMI 32.7 kg/m^2^		Intervention (n=39) Control: lifestyle booklet (n=39)		Digital tool: LivingWELL; a 12-week intervention consisting of 1 face-to-face session, 4 phone consultations, and web-based support to achieve 5% BW reduction. It included a personalized diet and physical exercise plan with behavioral techniques such as motivational interviewing and action planning.		Decreases in BW, BMI, and WC in the intervention group, but not in the control group.		Increases in daily MPA and daily steps (but not daily VPA) in the intervention group.	
	Bughin et al [52], 2021, France	Two-arm RCT; sample size: 50; mean age 52.2 years; 54% female participants; mean BMI 36.5 kg/m^2^		Telerehabilitation program (n=25) Usual care (n=25)		Digital tool: Telemouv app; a 12-week multicomponent intervention (PA, diet, and education) available on smartphones and a website. The PA program included endurance exercises (targeting 150 minutes of PA per week), muscle strengthening (with gradual increases in volume), and balance exercises.		Significant decreases in BF% and FM in both groups, with no significant changes in BW, BMI, or WHR. No significant between-group differences for any outcomes.		No significant within- or between-group change or difference in the 6-minute walk test.	
	Hebden et al [53], 2014, Australia	Two-arm RCT; sample size: 51; mean age 22.8 years; 81% female participants; mean BMI 27.3 kg/m^2^		mHealth intervention (n=26) Control (n=25)		Digital tool: ePASS; a 12-week mHealth program targeting key lifestyle behaviors associated with weight gain, delivered via smartphone apps, internet forums, text messages, and emails. The PA program included 30 minutes of moderate-intensity PA per day for general health and 60 minutes per day for weight management.		Significant decreases in BW and BMI in both groups; no significant between-group differences.		Significant increase in daily LPA (but not MVPA) in the intervention group.	
	Hong et al [54], 2022, Korea	Two-arm RCT; sample size: 31; mean age 79.9 years; 100% female participants; mean BF% 41.6		Intervention (n=12) Control (n=17)		Digital tool: Smartphone telepresence platform; a 12-week smartphone mirroring-based telepresence physical exercise program conducted at home with 3 sessions per week, primarily focused on resistance training (with gradual intensity increases), all based on international guidelines; while the control group performed the same program in person.		Significant decreases in BF% in both groups and in BW in the control group; no significant between-group differences.		Significant left-hand grip strength improvement in the intervention group and right-hand in the controls; no significant between-group differences.	
	Hurkmans et al [16], 2018, Belgium	Four-arm RCT; sample size: 81; mean age 45.0 years; 71.6% female participants; mean BMI 32.0 kg/m^2^		Face-to-face (n=28) App (n=30) Combined (n=22) Control (n=22)		Digital tool: Mobile Weight Loss App; 12-week weight loss programs using face-to-face, mobile, and combined approaches, offering physical exercise and dietary guidance aligned with standard guidelines, along with personalized exercise plans provided by a coach.		Significant decreases in BMI in the face-to-face, app, and combined groups; no significant difference between the 3 interventions.		Significant improvements in MVPA; no significant between-group differences.	
	Johnson et al [55], 2019, United States	Three-arm RCT; sample size: 30; mean age 43.2 years; mean BMI 36.1 kg/m^2^		Videoconferencing (n=10) In-person (n=10) Control (n=10)		Digital tool: Withings app and Healow app; 12-week telemedicine-based health coaching, including physical exercise routines, goal setting, and PA progression, with feedback provided by a registered dietitian and exercise physiologist. PA program followed international guidelines recommending at least 150 minutes of PA per week (30 minutes of MVPA, 5 days per week).		Significant differences in BW reduction between the study groups, with greater reduction in the videoconferencing group; no significant between-group differences in BMI. Decrease in BW and BMI in all groups.		Improvement in daily steps in all groups, with a significantly greater improvement in the videoconferencing group.	
	Pressler et al [56], 2010, Germany	Two-arm RCT; sample size: 105; median age 48 years; 11.8% female participants; mean BMI 29.0 kg/m^2^		Structured web-based exercise (n=66) Nonstructured web-based exercise (n=39)		Digital tool: Interactive internet platform; a 12-week intervention with structured or nonstructured internet-delivered physical exercise programs performed individually, alongside education sessions. The exercise program included 3 moderate endurance sessions and 1 strength training session per week.		Significant decreases in WC in both groups and in BMI and BF% in the control group; no significant between-group differences in any of these outcomes.		No significant changes in daily step counts in any study group.	
**Intervention duration: 8-10 weeks**											
	Ballin et al [57], 2020, Sweden	Two-arm RCT; sample size: 77; mean age 71.0 years; 50% female participants; mean BMI 29.2 kg/m^2^		Web-based exercise (n=38) Supervised exercise (n=39)		Digital tool: Healthy Ageing Initiative; a 10-week intervention consisting of a web-based progressive interval training program (10 weekly videos, 3 sessions per week), while the supervised exercise group participated in in-person sessions.		Significant decreases in BF% and FM in both groups, and in BMI only in the supervised exercise group; no significant between-group differences, except for FM (with a greater reduction in the supervised exercise group).		—	
	Hyun [58], 2021, Korea	Two-arm RCT; sample size: 16; mean age 38.4 years; 100% female participants; mean BMI 25.6 kg/m^2^		Moderate intensity training (n=8) High intensity training (n=8)		Digital tool: Real-time video web program; a 8-week real-time video web program based on international PA and exercise guidelines, including bidirectional communication along with instructor feedback. Training sessions lasted 30 minutes (for moderate intensity group) or 50 minutes (for high intensity group), held 3 times weekly, with exercise intensity increasing biweekly for the high intensity group.		Significant decreases in BW, WC, FM, BMI, and BF% in both groups, with significant between-group differences for BMI, WC, and FM (greater reductions in the high intensity group), but not for BW or BF%.		Significant left-hand grip strength improvement in the high intensity group and right-hand in both groups; significant between-group differences.	
	Seo et al [59], 2023, Korea	Three-arm RCT; sample size: 75; mean age 48.3 years; 100% female participants; mean BMI 25.5 kg/m^2^		Virtual reality exercise (n=25) Indoor bicycle exercise (n=25) Control (n=25)		Digital tool: VRFit app; an 8-week virtual reality physical exercise program using an Internet of Things sensor attached to an indoor bicycle, connected to a smartphone, and paired with a head-mounted display for immersive virtual reality exercise. Exercise sessions were conducted 3-5 times weekly, following international guidelines.		Significant decreases in BMI in the virtual reality group, with significant between-group differences (no BMI change in the 2 other groups).		Significant improvement in exercise fun in the virtual reality group, along with significant between-group differences.	

^a^PA: physical activity.

^b^RCT: randomized controlled trial.

^c^BW: body weight.

^d^BF%: body fat percentage.

^e^FM: fat mass.

^f^LPA: light physical activity.

^g^MVPA: moderate to vigorous physical activity.

^h^mHealth: mobile health.

^i^Not available.

^j^WC: waist circumference.

^k^WHR: waist-to-hip ratio.

^l^MPA: moderate physical activity.

^m^VPA: vigorous physical activity.

### Qualitative Data Synthesis

The duration of the interventions ranged from 8 weeks to 24 months, with an average of 6.6 (SD 5.5) months. Although all studies included interventions involving PA or exercise through action planning, coaching, or goal setting, there were substantial variations in the PA-related methods and techniques used, as well as in the inclusion of other lifestyle components alongside PA. A total of 14 studies [18,35-38,41,44,50,52-54,56,57,59] used digital interventions that delivered structured physical exercise programs, while other studies included flexible PA or exercise routines based on international guidelines. Regarding the type of physical exercise, all studies incorporated aerobic or moderate to vigorous PA (MVPA) as a core component, while 13 studies [18,35,36,40-42,44,50,52-54,56,57] additionally included resistance or strength training. All but 3 studies [44,56,59] incorporated multiple lifestyle components, including diet, sleep, and stress management, with diet being the most commonly used component alongside PA. Dietary guidance was provided in the form of dietary plans, goal setting based on dietary guidelines, nutritional advice, dietary coaching, and behavior change strategies related to eating habits.

Various digital tools, including mobile apps, websites, wearable devices, telecommunication platforms (eg, videoconferencing), virtual reality, or a combination of 2 or more in most studies, were used in the interventions. These tools supported features such as program delivery, coaching, feedback provision, goal setting, tracking, and progress monitoring. They were implemented in various ways, including automated or manual feedback, self-guided or professionally guided programs, real-time or asynchronous coaching, gamification elements, integration with other health platforms, and different levels of interactivity. This resulted in a diverse range of intervention designs, delivery modes, and user engagement levels.

While 4 studies [35,36,41,47] used a single-arm design, the control groups in the other studies exhibited considerable variability, including active controls (such as alternative digital or nondigital interventions or usual care) [33,34,37-40,42,43,45,46,49,51-58], passive control (such as no intervention or waitlist group) [20,44,48], or a combination of both [16,18,50,59], reflecting a diversity in methodological designs and approaches. All single-arm design studies demonstrated a significant reduction in outcome variables. Similarly, interventional studies comparing digital interventions to passive controls showed favorable effects. However, in studies comparing experimental groups to nondigital active controls, the results were inconsistent, with some reporting greater reductions in outcome variables following digital interventions [37,42,43,45,49,51,59], while others found no significant difference [16,18,38-40,46,50], and one [34] even reported less reduction following digital interventions. Common nondigital interventions included educational booklets, self-care advice, and in-person physical exercise programs.

Among the studies included in this review, 25 evaluated BW as an outcome variable. Overall, 22 studies (with an average intervention duration of 6.5, SD 4.9 months, ranging from 8 weeks to 24 months) reported significant BW reduction (range −1.3 to −8.4 kg) following digital intervention programs [18,20,33,35-43,45-51,53,55,58], while 3 studies found no significant change in BW following a 12-week multicomponent telerehabilitation program [52], a 12-week smartphone mirroring-based telepresence exercise program [54], or a 24 months of mobile health (mHealth) intervention [34]. A total of 10 studies (with an average intervention duration of 9.0, SD 8.4 months, ranging from 3 to 24 months) found significant differences in postintervention BW between their study groups [33,34,37,42,43,45,48-50,55], most of which used active control groups, with one study [48] using passive controls and another [50] using a combination of both. However, 10 studies (with an average intervention duration of 4.7, SD 1.7 months, ranging from 8 weeks to 6 months) reported no significant between-group differences in BW [18,20,38-40,46,52-54,58], most of which used active control groups, with one study [20] using passive controls and another [18] using a combination of both. No clear pattern was observed in BW outcomes when comparing experimental groups to nondigital active controls, with some studies reporting greater BW reduction following digital interventions [37,42,43,45,49], while others found no significant difference [18,38-40,46] or reported less BW reduction following digital interventions [34].

In this review, 23 studies examined BMI as an outcome measure. In total, 19 studies (with an average intervention duration of 5.9, SD 5.2 months, ranging from 8 weeks to 24 months) observed a significant reduction in BMI (range −0.4 to −3.4 kg/m^2^) following digital intervention programs [16,20,33,36,37,39,41,42,45-51,53,55,58,59], while 4 studies (with an average intervention duration of 5.1, SD 4.6 months, ranging from 10 weeks to 12 months) reported no significant BMI change following digital interventions [35,52,56,57]. A total of 8 studies (with an average intervention duration of 4.6, SD 3.4 months, ranging from 8 weeks to 12 months) reported significant differences in postintervention BMI between study groups [37,42,45,48-50,58,59], most of which used active control groups, while one study [55] used passive controls and 2 others [50,59] used a combination of both. However, 9 studies (with an average intervention duration of 6.3, SD 6.8 months, ranging from 10 weeks to 24 months) found no significant between-group differences in BMI changes [16,20,33,39,46,52,55-57], most of which used active control groups, with one study [20] using passive controls and another [16] using a combination of both. No clear trend was observed in BMI outcomes when comparing experimental groups to nondigital active controls, with some studies reporting greater BMI reduction following digital interventions [37,42,45,49,59], while others found no significant difference [16,39,46].

A total of 16 studies examined WC as an outcome measure. Overall, 15 studies (with an average intervention duration of 5.7, SD 3.0 months, ranging from 8 weeks to 12 months) found a significant reduction in WC (range −2.1 to −9.2 cm) following digital intervention programs [18,20,35,37-39,41,42,44,46,48,50,51,56,58], while one study [49] showed no significant change following a 3-month multicomponent lifestyle intervention delivered via a mobile app. In total, 8 studies (with an average intervention duration of 5.3, SD 3.1 months, ranging from 8 weeks to 12 months) reported significant between-group differences in postintervention WC [18,37,42,44,48-50,58]; however, 5 studies (with an average intervention duration of 5.4, SD 1.3 months, ranging from 12 weeks to 6 months) indicated no significant between-group differences [20,38,39,46,56]. There was also no clear trend in WC outcomes when comparing experimental groups to nondigital active controls, with 3 studies [37,42,49] reporting greater WC reduction following digital interventions, while 3 others [38,39,46] found no significant difference. WHR was assessed in 6 studies, with 2 studies (conducting 6-month and 3-month interventions) reporting a significant reduction in WHR (−0.02 and −0.01, respectively) [38,50], while the other 4 studies (with an average intervention duration of 6.0, SD 4.2 months, ranging from 12 weeks to 12 months) found no change in WHR following digital intervention programs [36,41,49,52].

In this review, FM was examined as an outcome measure in 7 studies (with an average intervention duration of 7.9, SD 7.8 months, ranging from 8 weeks to 24 months), all of which found a significant reduction (range −0.4 to −6.5 kg) following digital intervention programs [20,33,35,46,52,57,58]. Among them, 3 studies (with an average intervention duration of 3.5, SD 2.2 months, ranging from 8 weeks to 6 months) reported significant between-group differences after the intervention [20,57,58], while 3 others (with an average intervention duration of 11.0, SD 11.3 months, ranging from 12 weeks to 24 months) found no significant difference in FM between their study groups [33,46,52]. In terms of BF%, 9 studies measured this outcome (with an average intervention duration of 6.8, SD 7.1 months, ranging from 8 weeks to 24 months), all of which found a significant reduction (range −0.3% to −4.1%) from pre- to posttests in digital intervention groups [20,33,36,46,52,54,56-58]. However, the majority (8 studies) reported no significant between-group differences in BF% changes, and one study [36] followed a single-arm design. No clear trend was observed in FM and BF% outcomes when comparing digital-based experimental groups to nondigital active controls.

Regarding PA, 8 studies provided information on changes in MVPA following digital interventions, with 4 reporting significant improvements with an average intervention duration of 9.2 (SD 9.9; range 3-24) months [16,33,43,47] and the other 4 observing no change in MVPA with an average intervention duration of 5.2 (SD 1.5) months [18,20,39,53]. A similar trend of inconsistent results was observed across other PA-related variables, including general PA [37,45,48-50], light PA [18,33,53], moderate PA [20,51], vigorous PA [20,51], daily steps [51,55,56], grip strength [41,54,58], and the 6-minute walk test [41,52]. In addition, 5 other variables, including exercise capacity [44], exercise fun [59], exercise self-efficacy [49], sit-to-stand test [41], and gait speed [41], were each assessed in just one study. Inconsistent trends in postintervention changes were observed in these variables, with some showing significant improvements [44,49,59], while others displayed no significant changes [50], and some revealed mixed results within individual studies [41,54].

### Meta-Analysis

**Figure 2:**
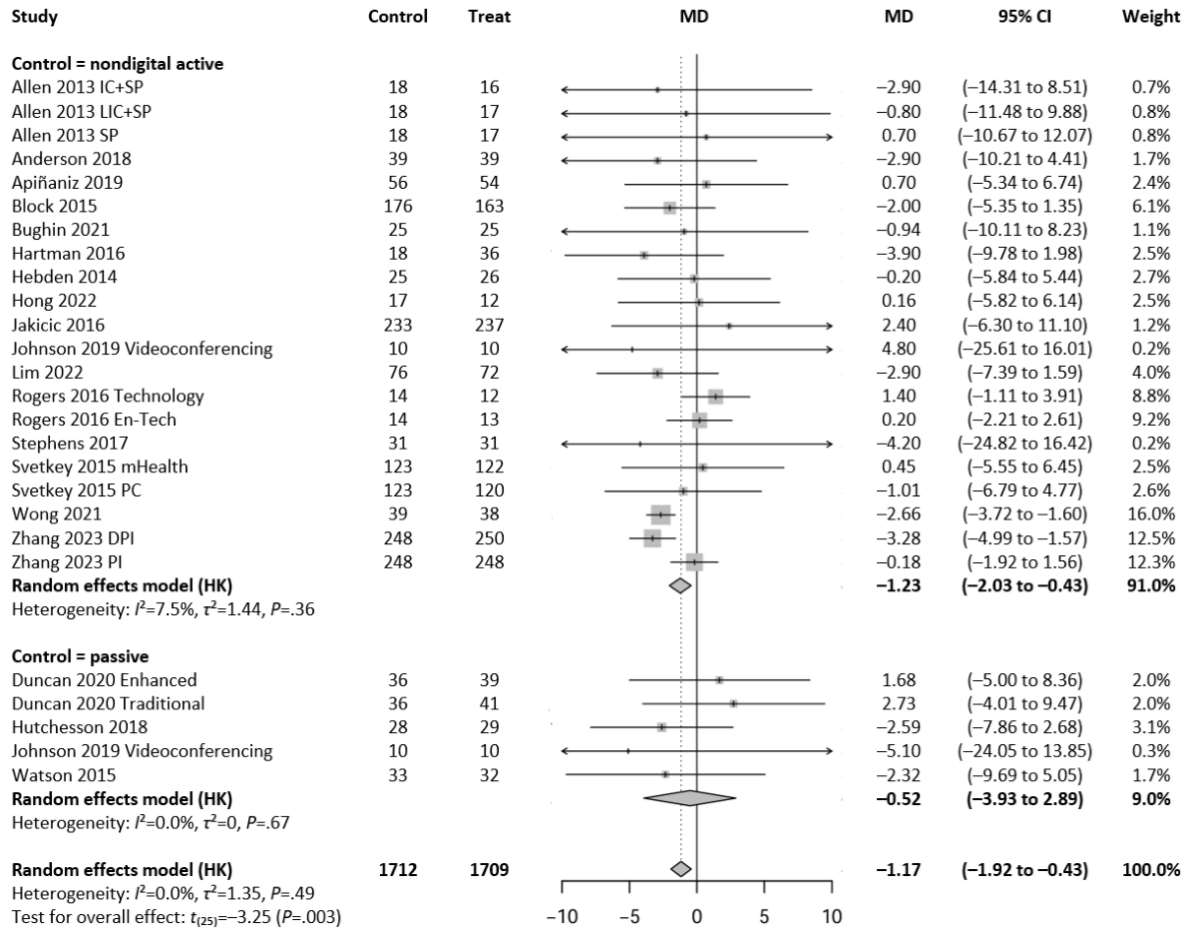
Forest plot showing the effect of guideline-based digital exercise interventions on body weight across 26 comparisons between digital exercise interventions and control conditions, using a random-effects model with Hartung-Knapp (HK) adjustment. Subgroup analyses are presented for comparisons with nondigital active controls (21 comparisons) and passive controls (5 comparisons), while the last model represents a pooled analysis of all studies (n=1709 intervention participants vs n=1712 controls). Each horizontal line represents a study’s 95% CI, with diamond shapes reflecting the pooled estimates. DPI: diet and physical activity intervention; En-Tech: enhanced technology; IC: intensive counseling; LIC: less intensive counseling; MD: mean difference; mHealth: mobile health; PC: personal coaching; PI: physical activity intervention; SP: smartphone.

A total of 26 intervention arms from 19 independent studies were included in the meta-analysis assessing the effect of digital exercise interventions on BW. The overall pooled analysis revealed a significant reduction in BW following digital exercise interventions compared to control conditions (MD=−1.17 kg, 95% CI −1.92 to −0.43; *P*=.003; t_25_=−3.25). Heterogeneity was not significant (*I*^2^=0.0%; *τ*^2^=1.35; *P*=.49), indicating consistency across studies. Subgroup analyses based on control type showed a greater effect in studies comparing digital interventions to active (nondigital) controls than to passive controls (MD=−1.23 kg vs −0.52 kg, respectively). Figure 2 [18,20,33,34,37,39,40,42,43,45,46,48-55] shows the results of the meta-analysis assessing the effect of guideline-based digital exercise interventions on BW.

**Figure 3:**
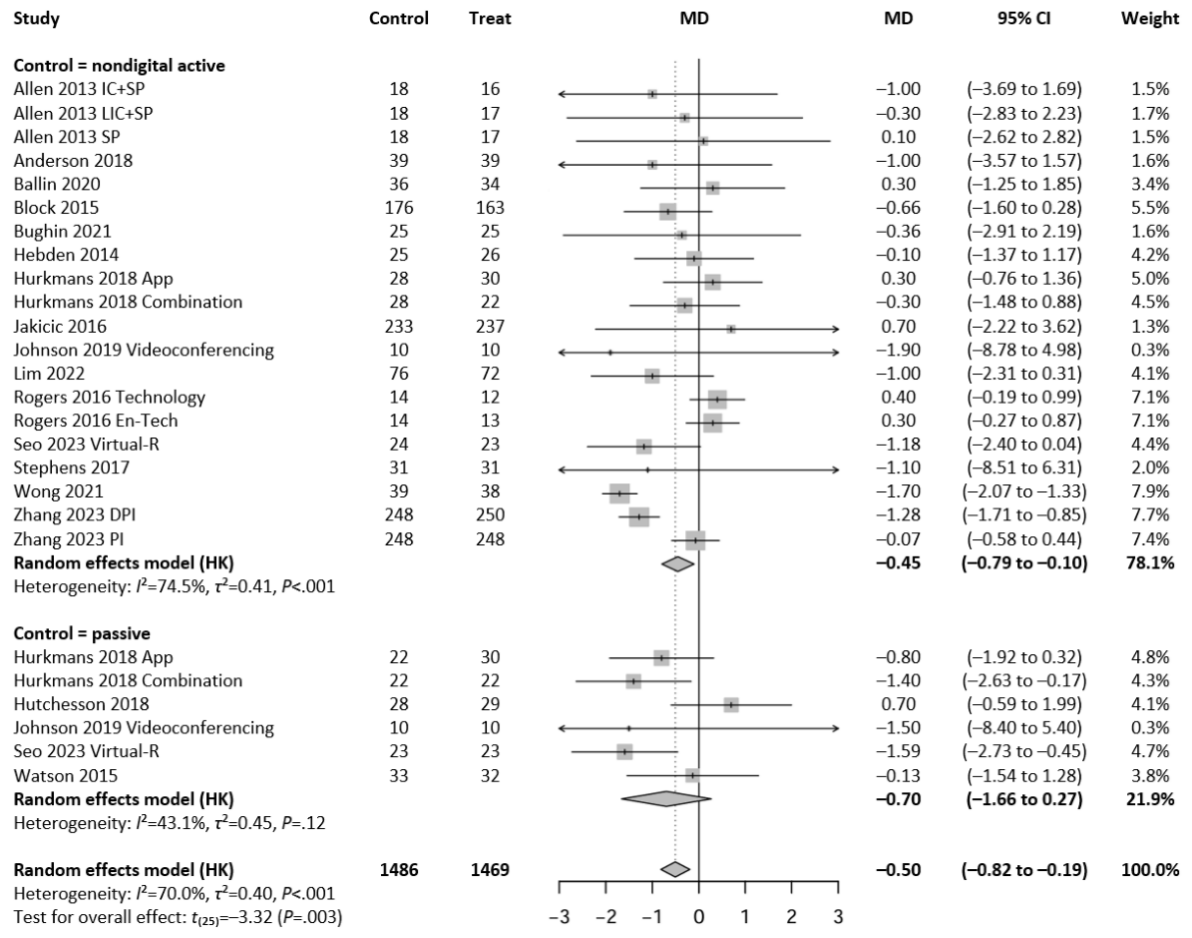
Forest plot showing the effect of guideline-based digital exercise interventions on BMI across 26 comparisons between interventions and control conditions, using a random-effects model with Hartung-Knapp (HK) adjustment. Subgroup analyses are presented for comparisons with nondigital active controls (20 comparisons) and passive controls (6 comparisons), while the last model represents a pooled analysis of all studies (n=1469 intervention participants vs n=1486 controls). Each horizontal line represents a study’s 95% CI, with diamond shapes reflecting the pooled estimates. DPI: diet and physical activity intervention; En-Tech: enhanced technology; IC: intensive counseling; LIC: less intensive counseling; MD: mean difference; PI: physical activity intervention; SP: smartphone only; Virtual-R: virtual reality.

Regarding BMI, a total of 26 intervention arms from 17 independent studies were included in the meta-analysis. A random-effects model revealed a statistically significant reduction in BMI favoring digital interventions (MD=−0.50 kg/m^2^, 95% CI −0.82 to −0.19; *P*=.003; t_25_=−3.32), with significant heterogeneity across studies (*I*^2^=70.0%; *τ*^2^=0.40; *P*<.001). Subgroup analyses based on control type showed a greater effect in studies comparing digital interventions to passive controls than to active (nondigital) controls (MD=−0.70 kg/m^2^ vs −0.45 kg/m^2^, respectively). Figure 3 [16,20,33,37,39,42,45,46,48-53,55,57,59] shows the results of the meta-analysis assessing the effect of guideline-based digital exercise interventions on BMI.

**Figure 4:**
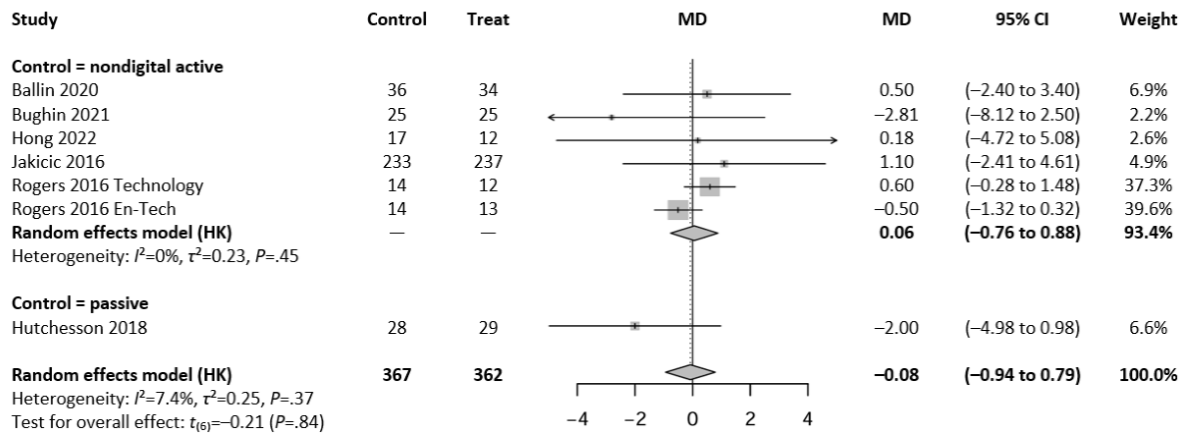
Forest plot showing the effect of guideline-based digital exercise interventions on body fat percentage using a random-effects model with Hartung-Knapp (HK) adjustment. Subgroup analyses are presented for comparisons with nondigital active controls (6 comparisons), while only 1 comparison was available for passive controls. The last model represents a pooled analysis of all studies (n=362 intervention participants vs n=367 controls). Individual study estimates are shown with 95% CIs; diamonds represent pooled estimates. En-Tech: enhanced technology; MD: mean difference.

Regarding BF%, a total of 7 intervention arms from 6 independent studies were included in the meta-analysis. The overall analysis revealed no statistically significant difference between interventions and control conditions (MD=−0.08%, 95% CI −0.94 to 0.79; *P*=.84, *t*_6_=−0.21). Heterogeneity was not significant (*I*^2^=7.4%; *τ*^2^=0.25; *P*=.37). Figure 4 [20,33,46,52,54,57] shows the results of the meta-analysis assessing the effect of guideline-based digital exercise interventions on BF%.

### Risk of Bias

**Figure 5:**
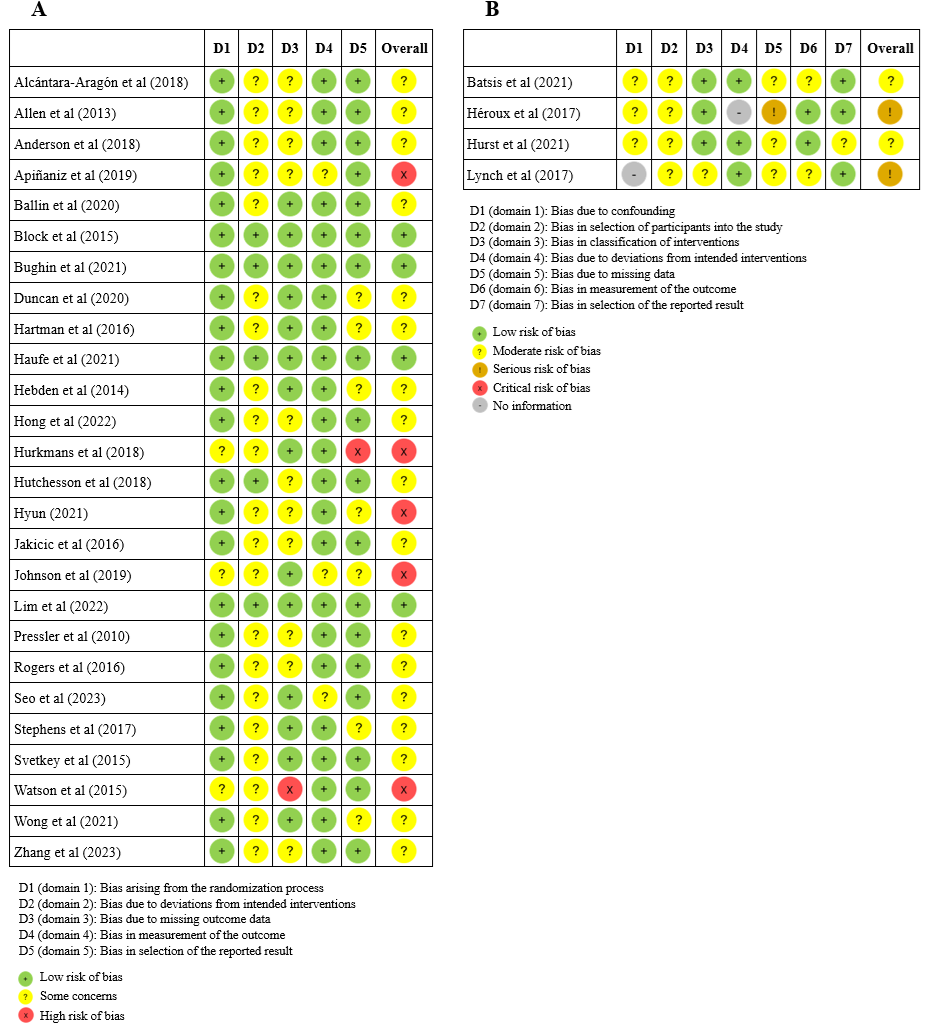
Summary of risk of bias assessment using (A) the Cochrane Risk of Bias (version 2) tool for randomized controlled trials and (B) the Cochrane Risk of Bias in Nonrandomized Studies of Interventions tool for nonrandomized studies.

Figure 5 [16,18,20,31-59] summarizes the risk of bias assessment for the included studies, with panel A presenting the overall assessment for RCTs and panel B for nonrandomized studies. Among the 26 RCTs, 4 (15%) were classified as having a “low risk of bias,” indicating a strong methodological design with minimal threats to validity. In total, 65% (17/26) of the studies were rated as having “some concerns,” suggesting potential sources of bias that could affect the reliability of their findings. In addition, 19% (5/26) of the studies were deemed to have a “high risk of bias,” highlighting significant methodological limitations that may compromise the robustness of their results. Among the 4 non-RCTs, 2 (50%) were found to have a “moderate risk of bias,” implying methodological shortcomings that could impact their conclusions. The remaining 50% (2/4) of the studies were rated as having “some concerns,” indicating the presence of potential biases that warrant caution when interpreting their findings. Excluding studies with a high or serious risk of bias did not substantially alter the overall pattern of results. Similarly, findings from the 4 low-risk-of-bias studies [42,44,45,52] aligned with the general patterns, and the 7 studies with high or serious risk of bias showed the same pattern [16,35-37,40,55,58].

## Discussion

### Principal Findings

This systematic review and meta-analysis assessed the effectiveness of guideline-based digital exercise interventions in improving body composition in adults with overweight and obesity, aiming to identify gaps in the literature and recommend strategies for developing and implementing future digital health interventions. In addition, alterations in PA were considered as a secondary outcome. The main findings of the meta-analysis were (1) guideline-based digital exercise interventions significantly reduced BW (MD=−1.17 kg; *P*=.003), with greater effects observed when compared to active (nondigital) controls; (2) BMI was also significantly reduced (MD=−0.50 kg/m^2^; *P*=.003), with stronger effects observed in studies using passive (waitlist) control groups; and (3) no significant effect was found for BF% (MD=−0.08%; *P*=.84). The main qualitative findings were (1) digital exercise intervention programs led to significant reductions in BW (22/25, 88% studies; range −1.3 to −8.4 kg); BMI (19/23, 83% studies; range −0.4 to −3.4 kg/m^2^); WC (15/16, 94% studies; range −2.1 to −9.2 cm); BF% (9/9, 100% studies; range −0.3% to −4.1%); and FM (7/7, 100% studies; range −0.4 to −6.5 kg); (2) inconsistent results were observed for WHR and PA outcomes; (3) no clear trend was identified between intervention duration or type and improvements in outcome variables; and (4) substantial variations were observed in intervention designs, comparators, confounders, and participant characteristics. The digital exercise apps and tools included in this review addressed behavior change through multiple evidence-based strategies grounded in established health behavior theories. Most interventions incorporated goal setting, self-monitoring, and feedback loops, known as core components of behavior change techniques. For instance, studies using apps such as Lose It!, Alive-PD, MetS App, and Balanced App featured personalized goal setting for PA, daily exercise reminders, and tracking tools to enhance user accountability and adherence [18,42,48,49]. Several interventions explicitly applied behavioral theories, such as social cognitive theory, the transtheoretical model, and motivational interviewing, emphasizing constructs such as self-efficacy, stages of readiness, and problem-solving [20,34,45,51]. These constructs were operationalized through coaching, interactive feedback, and gradual PA progression plans. In this context, evidence indicates that human-computer interaction plays a vital role in the effectiveness of digital health interventions, as well-designed interfaces enhance user engagement and adherence through personalized feedback, interactivity, and intuitive navigation [60]. Moreover, some studies used peer interaction or professional coaching via telehealth, which are known to enhance engagement in PA [33,37,47]. Together, these elements show that digital exercise apps, when grounded in PA guidelines and behavior change theory, may extend beyond the delivery of exercise routines to potentially support sustained behavior change by addressing key psychological and social determinants of health behavior.

### Body Composition Outcomes

Consistent with this review, evidence from other review studies suggests that mHealth and digital tools can be effective for BW reduction [61-65]. Results from a systematic review [66] indicate that eHealth interventions are generally more effective than no care or usual care interventions, aligning with this meta-analysis, which found a significant average BW loss of approximately 1.17 kg associated with digital exercise interventions. This effect remained significant when compared to active (nondigital) control interventions (MD=−1.23 kg), suggesting that digital platforms may have advantages over traditional, face-to-face programs. These advantages may stem from features such as improved accessibility, personalization, and real-time feedback, all of which can contribute to greater user engagement. Research suggests that the impact of digital interventions on BW reduction may be influenced by factors such as intervention duration and design. In particular, the effectiveness of mHealth interventions for BW reduction tends to increase with longer intervention durations [63,67]. Furthermore, it has been documented that digital interventions incorporating personalized feedback and human interaction are more effective than fully automated programs [66,68]. In this regard, evidence suggests that successful digital interventions often include components such as self-monitoring, social support, goal setting, and prolonged engagement, all of which are crucial for enhancing user engagement and promoting sustained behavior change [69,70].

In line with the results of this review, evidence from comparable review studies suggests that mHealth and digital interventions can be effective in reducing BMI [61-63,71,72], with their impact largely influenced by factors such as the type of intervention, its integration with other health services, and user engagement levels [66,73,74]. In this review, no clear pattern was found between the type and duration of interventions and BMI reductions. However, a stronger effect was observed in studies using passive (waitlist) control groups compared to active controls. A review [63] found that longer intervention duration enhanced the effectiveness of mHealth and digital interventions in reducing BMI. Cultural and regional factors may also indirectly influence the effectiveness of mHealth interventions in BW outcomes; nevertheless, a study evaluating a digital weight loss intervention across multiple countries [75] found similar BW outcomes although levels of participants’ engagement varied. These factors may contribute to the high degree of heterogeneity among studies on this topic, as their inclusion adds complexity to the interventions. However, it is important to note that the methodological quality of studies on digital interventions for BW and BMI reduction varies significantly, which can affect the reliability of their findings and the associated review studies [66,76], although excluding studies with low methodological quality did not substantially alter the overall pattern of results in this study.

WC and WHR are 2 important obesity-related measurements used to assess fat distribution. A meta-analysis [61] reported a statistically significant reduction in WC in adults with overweight and obesity following mHealth interventions. Similarly, another review study [72] found a consistent reduction in WC following web- and mobile-based interventions. These findings align with the results of this review, which examines the effectiveness of guideline-based digital exercise interventions on WC. Results from another systematic review [77] show that mHealth and eHealth interventions, particularly those with personalized feedback, have been associated with reductions in central obesity measures. While there is a lack of similar review studies in the literature assessing the impact of digital interventions on WHR, this review identified 2 studies reporting a reduction in WHR, while 4 other studies reported no change following digital intervention programs. This insignificant trend, which has not been observed in BW, BMI, or WC, may be attributed to the fact that WHR appears to be an inaccurate measure of obesity in individuals with obesity, as both waist and hip circumferences reflect fat accumulation in these individuals [78], and fat loss occurs throughout the body, not in specific regions.

Consistent with the qualitative findings of this systematic review, several studies [63,72] have reported that mHealth and digital interventions can lead to significant reductions in BF%. However, this meta-analysis found no statistically significant difference in the change in BF% between intervention and control groups, with subgroup analysis also revealing no significant effect when compared to active controls. This may be attributed to the limited number of studies assessing BF%, highlighting the need for further research. Consistently, results from a meta-analysis show an insignificant change in BF% following mHealth interventions in adults with overweight and obesity, while FM reduction was significant [61]. The discrepancy in BF% but not in FM, compared to the findings of this review, may be due to the primary focus of this review on the PA component, specifically guideline-based PA, as physical exercise contributes to increased lean mass, thereby having a greater impact on BF%. Research shows that interventions using telehealth and text messaging have also demonstrated positive effects on body fat reduction by providing continuous support and motivation, which can enhance adherence to the lifestyle changes necessary for fat reduction [79]. The use of multiple electronic modalities, such as combining mHealth with other digital tools, tends to yield more significant results in fat reduction compared to single-modality interventions, offering a more comprehensive and engaging intervention experience [80]. Although mHealth and digital interventions demonstrate potential for fat reduction, evidence indicates that the long-term sustainability of these interventions remains uncertain, and their effectiveness may differ based on factors such as socioeconomic status and ethnicity, which could contribute to disparities in health outcomes [81]. Overall, based on the available literature and the present findings, effective digital PA interventions for improving body composition typically incorporate human interaction and sustained engagement, with personalized feedback, social support, and goal setting further enhancing their efficacy.

### PA Outcomes

There is limited evidence showing apps to be more effective for PA outcomes than face-to-face interventions [61,82,83]; however, digital tools may offer benefits in terms of cost-effectiveness and the ability to reach a larger and more geographically dispersed population [10,11]. Insufficient and inconsistent results were found for PA outcomes in this review. However, reports from other review studies indicate an increase in general PA [84], daily step counts [65], and weekly MVPA [65] following mHealth interventions. Another review study [85] examining the effects of mHealth interventions on older adults reported an increase in weekly MVPA. A recent umbrella review of mHealth and eHealth interventions [86] reported a significant increase in daily step counts among adult populations. However, 2 systematic reviews found no significant change in general PA [82] or MVPA [61] following mHealth interventions. These discrepancies, which have also been observed in this review, may be explained by variations in intervention design and duration, target populations, and the methods used to measure PA outcomes. A review study [83] highlighted that combining digital media with face-to-face support showed the greatest potential for increasing PA compared to digital-only interventions. This highlights the need for more rigorous research to confirm the long-term benefits of digital interventions in improving PA; however, the methodological quality of the included studies in this review did not alter the results related to PA outcomes.

### Limitations and Strengths

This systematic review contains limitations that need to be acknowledged. While the variability in intervention designs and duration has made direct comparisons challenging, variations in participant characteristics (such as baseline BMI, PA levels, sex distributions, age, socioeconomic status, and comorbidities) may have influenced individual study results, and consequently, the findings of this systematic review. In addition, it is important to elaborate on the potential confounding variables that may have influenced the results across the included studies. These confounders, often unevenly distributed or poorly controlled across trials, can influence both the internal and external validity of the findings. For instance, dietary intake, a key determinant of physical and metabolic outcomes, was not consistently reported or controlled in many studies, making it difficult to isolate the effects of the mHealth interventions alone. Similarly, factors such as motivation, digital literacy, and health literacy can substantially influence both adherence and outcomes but were rarely measured or adjusted for. In some populations, cultural factors may also affect attitudes toward PA and technology use, further complicating comparisons across studies conducted in different geographic or cultural contexts. These unmeasured or uncontrolled confounding variables introduce heterogeneity and potential bias into the pooled results, and their presence highlights the need for better standardization and reporting in future mHealth intervention studies. The quality of evidence supporting mHealth interventions should also be considered, as it may vary widely, although this was not observed in this study. Previous systematic reviews and meta-analyses [87,88] have rated the methodological quality of mHealth studies as low to moderate, with only a few achieving high-quality ratings, primarily due to differences in study design, sample size, and outcome measures.

Despite the limitations, this systematic review presents a comprehensive evaluation of guideline-based digital exercise interventions across multiple anthropometric and body composition outcomes, as well as PA-related variables. The inclusion of a variety of intervention formats allows for a broad understanding of the effectiveness of mHealth and digital approaches. In addition, the review follows a rigorous methodology, ensuring high-quality data analysis and synthesis, thereby contributing to the growing body of evidence-based literature on digital health interventions for improving body composition and promoting PA.

Overall, this review provides insights for individuals, trainers, coaches, health care providers, health organizations, app developers, and policy makers by identifying the potential benefits of apps and digital tools focusing on PA for BW management, thereby contributing to the broader effort to manage overweight and obesity effectively. In addition, the findings highlight areas for future research to optimize the design and implementation of fitness apps for better health outcomes. Future research should focus on the role of personalized and adaptive digital programs, as well as their integration with other lifestyle interventions. By understanding the essential characteristics that enhance the effectiveness of digital health interventions, stakeholders can make informed decisions about integrating them into weight management programs and public health strategies. Meanwhile, clinicians and health care providers can leverage digital technologies to improve accessibility, enhance patient engagement, optimize treatment adherence, and support long-term success in weight management programs.

### Conclusions

This study highlights the potential of guideline-based digital exercise interventions for addressing overweight and obesity. While significant reductions in BW and BMI were observed, results were less clear regarding body composition changes. Although longer-duration interventions showed a trend toward greater BW reduction, no clear pattern emerged between the type or duration of interventions and improvements in body composition. Key characteristics that are often identified as critical for successful digital PA interventions include personalized content, regular user engagement, and integration with other health strategies, such as dietary management and social support. While this review highlights the potential benefits of guideline-based digital exercise interventions for weight management, it also provides valuable insights to inform decisions about integrating them into health programs and public health strategies. Overall, digital health interventions can reach a larger population at a lower cost and have been shown to be effective for weight loss; however, evidence of their superiority over traditional methods is limited and inconsistent, suggesting that digital interventions may serve as a complementary approach rather than a definitive replacement for traditional methods. As technology continues to evolve, future interventions have the potential to examine and offer more tailored, engaging, and effective solutions for weight management.

## Appendix

Multimedia Appendix 1 Keywords used in the database search with Boolean operators: AND (between categories) and OR (between keywords within each category).

Multimedia Appendix 2 PRISMA 2020 checklist.

## Abbreviations

BF%: body fat percentage
BW: body weight
FM: fat mass
MD: mean difference
MeSH: Medical Subject Headings
mHealth: mobile health
MVPA: moderate to vigorous physical activity
PA: physical activity
PRISMA: Preferred Reporting Items for Systematic Reviews and Meta-Analyses
RCT: randomized controlled trial
WC: waist circumference
WHO: World Health Organization
WHR: waist-to-hip ratio
