# Supplementary material for: Guideline-Based Digital Exercise Interventions for Reducing Body Weight and Fat and Promoting Physical Activity in Adults With Overweight and Obesity: Systematic Review and Meta-Analysis
Source: Interact J Med Res. 2025 Aug 7;14:e73656. doi: 10.2196/73656 (PMC12371298; doi:10.2196/73656)
Supplement: Multimedia Appendix 1 [file ijmr_v14i1e73656_app1.docx]

## Multimedia Appendix 1

Keywords used in database search with Boolean operators: AND (between categories) & OR (between keywords within each category)

|  | **AND** | | | | | |
| --- | --- | --- | --- | --- | --- | --- |
| **OR** | **Population** | **Population** | **App** | **Intervention** | **Outcomes** | **Design** |
|  | *adult*  *adults*  *adulthood*  *men*  *women*  *male*  *males*  *female*  *females*  *students*  *middle-aged*  *older*  *elderly*  *senior*  *employees*  *staff*  *workers* | *overweight*  *obesity*  *obese*  *excess weight*  *excessive weight*  *BMI*  *body fat*  *body mass index*  *adiposity* | *app*  *application*  *mobile*  *smartphone*  *smartwatch*  *remote*  *wearable*  *android*  *iOS*  *digital*  *platform*  *online*  *internet*  *mHealth*  *eHealth*  *web-based*  *app-based*  *electronic device* | *fitness*  *exercise*  *training*  *workout*  *sport*  *sports*  *aerobics*  *gym*  *physical activity*  *run*  *running*  *jogging*  *walking*  *yoga*  *stretching*  *cycling* | *body weight*  *body mass*  *weight loss*  *weight reduction*  *weight management*  *weight control*  *body composition*  *fat mass*  *fat percentage*  *adipose tissue*  *waist-hip*  *waist-to-hip*  *waist circumference*  *WHR*  *subcutaneous*  *body shape*  *body size*  *skinfold thickness*  *anthropometr** | *randomized*  *randomised*  *RCT*  *clinical*  *factorial*  *cluster*  *crossover*  *pre-test*  *pretest*  *baseline*  *post-test*  *follow-up*  *pre-post*  *parallel*  *intervention*  *interventional*  *quasi*  *controls*  *control* |
